# Honey as a Neuroprotective Agent: Molecular Perspectives on Its Role in Alzheimer’s Disease

**DOI:** 10.3390/nu17162577

**Published:** 2025-08-08

**Authors:** María D. Navarro-Hortal, Jose M. Romero-Márquez, Johura Ansary, Daniel Hinojosa-Nogueira, Cristina Montalbán-Hernández, Alfonso Varela-López, José L. Quiles

**Affiliations:** 1 Department of Physiology, Institute of Nutrition and Food Technology ‘‘José Mataix Verdú”, Biomedical Research Centre, University of Granada, Avda. del Conocimiento s.n., 18100 Armilla, Spain; 2 Department of Endocrinology and Nutrition, Virgen de las Nieves University Hospital, 18012 Granada, Spain; 3 Foundation for Biosanitary Research of Eastern Andalusia—Alejandro Otero (FIBAO), 18014 Granada, Spain; 4 Department of Specialized and Odontostomatological Clinical Sciences (DISCO), Università Politecnica delle Marche, Via Brecce Bianche, 60131 Ancona, Italy; 5 Unidad de Gestión Clínica de Endocrinología y Nutrición, Laboratorio del Instituto de Investigación Biomédica de Málaga (IBIMA), Hospital Universitario de Málaga (Virgen de la Victoria), 29590 Málaga, Spain

**Keywords:** manuka, tualang, antioxidant, polyphenols, amyloid, tau protein, acetylcholinesterase, neuroinflammation, oxidative stress

## Abstract

Alzheimer’s disease (AD) is the most prevalent form of dementia and a major global health challenge, characterized by progressive cognitive decline and neurodegeneration. Despite decades of research, there is currently no cure, and available treatments provide only limited symptomatic relief without halting disease progression. In this context, natural compounds with multi-targeted biological activities are being explored as potential complementary therapeutic strategies. Honey, a complex natural substance rich in bioactive phytochemicals, has emerged as a promising candidate due to its antioxidant, anti-inflammatory, anti-apoptotic, and enzyme-inhibitory properties. This review summarizes the molecular mechanisms underlying the neuroprotective effects of honey in the context of AD, with a particular focus on its ability to modulate oxidative stress, mitochondrial dysfunction, inflammation, apoptosis, β-amyloid accumulation, tau hyperphosphorylation, and neurotransmission-related enzymes. Notably, the botanical origin of honey significantly influences its composition and biological activity, as evidenced by studies on avocado, manuka, acacia, kelulut, chestnut, coffee, or tualang honeys. While preclinical findings are encouraging, especially in vitro and in invertebrate and rodent models, clinical validation is still lacking. Therefore, further research, including well-designed in vivo and human studies, is needed to clarify the therapeutic relevance of honey in AD. Overall, honey may represent a promising natural adjunct in the prevention or management of AD, but current evidence remains preliminary.

## 1. Introduction

Alzheimer’s disease (AD) is a progressive and lethal neurodegenerative disorder that represents the most prevalent form of dementia [1]. Dementias in general are the seventh cause of death globally and the second highest in high-income countries. According to the World Health Organization, more than 55 million people live with dementia worldwide and there are nearly 10 million new cases every year. Hence, the total cost of dementia worldwide was USD 1.3 trillion in 2019, and is expected to increase to USD 2.8 trillion by 2030 as the incidence is increasing [2]. Common AD early symptoms are memory problems like difficulty remembering recent conversations, names, or events. Other frequent manifestations are alterations in word finding, vision/spatial issues, and impaired reasoning or judgment, as well as apathy and depression. In advanced cases, the behavioral, cognitive, and motor declinations, including difficulty speaking, swallowing, and walking, interfere with the daily life of the patients, even incapacitating them. This leads to a high degree of disability and morbidity, which is converted into a severe challenge for society and the healthcare system. Therefore, all the problems associated with this pathology result in an important impact in physical, psychological, social, emotional, and economic terms [3].

AD is considered a complex and multifactorial disease in which the main molecular features are the synaptic dysfunction, neurotransmission alterations, protein misfolding, and other general aging-associated features such as oxidative stress, mitochondrial dysfunction, and inflammation [4]. Two of the most accepted hypotheses of the AD etiopathogenesis are the amyloid beta (Aβ) and the tau cascade, which are proteins with different locations reported as the main causes of the disease [4,5,6]. The Aβ plaques and the neurofibrillary tangles of hyperphosphorylated tau are deteriorative for cells and could lead to cell death or apoptosis, as well as to neuroinflammation and oxidative stress [4,5].

Although the number of dementia patients and the aged population are expected to grow, the disease incidence appears to have declined in recent years due to the improvement in risk factors such as the cardiovascular or educational level [7,8]. That fact suggests that the identification of and reduction in the risk factors may be an effective strategy. Currently, there is no preventive or curative treatment for AD [1]. The available treatments, which mainly consist of inhibitors of the acetylcholinesterase (AChE) enzyme, only provide a temporary and slight improvement of the symptoms in mid- to moderate-AD patients [3,9]. Thus, there is an overriding need to find or develop new tools for the prevention or management of this pathology. In this sense, one of the main modifiable risk factor is the diet [3], so bioactive compounds from foods could be an excellent approach. Foods present a wide and diverse composition of chemical compounds that may act on the hallmarks of the disease [9]. In that sense, apiculture products like honey are highlighted for their wealth in phenolic compounds, mainly flavonoids, that have been demonstrated to exert health benefits partly derived from their antioxidant and anti-inflammatory potential and, therefore, could be considered as a promising tool in the onset and development of neurodegenerative diseases like AD.

Based on all the above, the aim of this article is to summarize the knowledge about the impact of honey in AD in terms of the molecular aspects of the disease. A literature search was conducted in three major databases: PubMed, Scopus, and Web of Science (WoS) (Core Collection). The goal was to identify studies investigating the effects of honey or honey-derived extracts on AD or its core pathological mechanisms. To this end, the following search equation was applied: Honey AND (Alzheimer OR amyloid OR “tau protein” OR neuroinflammation OR apoptosis OR neurotransmission OR “oxidative stress” OR “mitochondrial dysfunction” OR “ion disbalance”). This combination of terms allowed for the inclusion of studies investigating at least one of the hallmark features of AD in the context of honey administration. The combined searches yielded the following results: PubMed: 646 records, Scopus: 1013 records, and WoS: 1712 records. After title and abstract screening, studies were excluded based on the following criteria: (i) review articles, (ii) studies unrelated to the nervous system or neurodegeneration, (iii) studies involving bees or bee venom, (iv) studies on apicultural products other than honey (e.g., royal jelly, bee pollen), (v) studies using honey mixed with additives without a honey-only control, (vi) non-English articles, and (vii) studies without accessible full text. After applying these criteria and removing duplicates, 27 unique articles were retained.

## 2. Phytochemical Characterization of Honeys

Honey is a natural product made by worker honeybees from honeydew or flower nectar [10,11,12]. It is collected by bees and transported into the hive, where concentration processes and enzymatic reactions take place to produce the honey. The composition and features of honey in terms of taste and color are linked to the type of flower source, climate, and geographical area, as well as processing methods and storage conditions [13]. This food contains a large variety of compounds, including water, sugars, proteins, minerals, vitamins, free amino acids, and phytochemicals [10,11]. Concerning the phytochemicals, the main classes of polyphenols present in this matrix are phenolic acids and flavonoids, mainly found as aglycones [13]. As mentioned, the phenolic content of honey varies notably depending on its botanical origin. Generally, lighter honeys, such as those produced from acacia, willow, ribwort, goldenrod, clover, rapeseed, sunflower, lime, thyme, and citrus flowers, tend to exhibit a lower concentration of total phenolic compounds when compared to darker varieties like chestnut, heather, forest honeydew, and buckwheat honey [14]. Among the phenolic acids most frequently identified in honey are p-coumaric, ferulic, caffeic, cinnamic, syringic, gallic, and chlorogenic acids. Regarding flavonoids, the dominant classes present include flavones, flavanols, and flavonols. Specific compounds commonly detected in honeys include quercetin, luteolin, apigenin, kaempferol, pinobanksin, chrysin, and pinocembrin. Information about the presence of phenolic compounds in different honeys is collected in Table 1 [11,14,15,16,17,18,19,20,21,22,23,24,25,26,27,28].

These compounds are considered key contributors to honey’s antioxidant properties, primarily due to their capacity to neutralize free radicals by generating more stable and less harmful molecular forms [11]. Antimicrobial, antifungal, antiviral, anti-inflammatory, anticancer, antidiabetic, antilipidemic, and protective effects on cardiovascular, nervous, respiratory, and gastrointestinal systems are some of the health benefits demonstrated by honey, supporting its use in the treatment of various disorders [10,11]. Even more, the benefits of honey in terms of oxidative stress, mitochondrial dysfunction, and inflammation, among others, mean it is considered as a promising tool in the management of healthy aging [29].

## 3. Effects of Honey on AD Features

**Figure 1:**
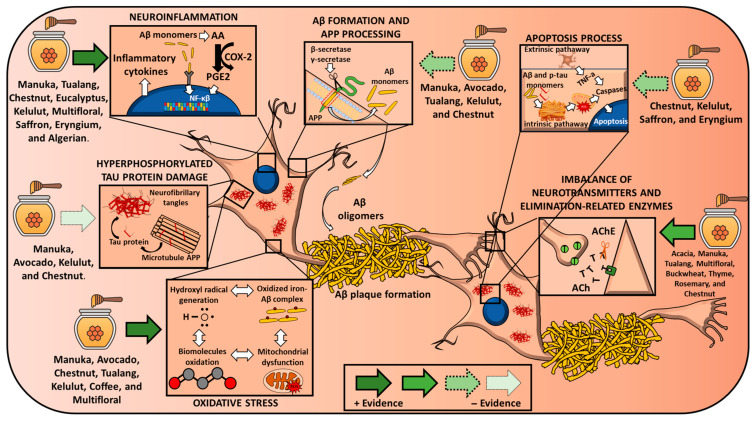
Effects of different honey types on the main molecular features of Alzheimer disease. Arrows’ design reflects the strength of the evidence. Abbreviations: AA = arachidonic acid; Aβ= amyloid beta; APP= amyloid precursor protein; AChE = acetylcholinesterase; COX-2 = cyclooxygenase-2; PGE_2_ = prostaglandin E_2_.

As mentioned above, honey exerts beneficial effects against the main pathological mechanisms of AD. The evidence regarding the effects of different types of honey on AD-related features is summarized below and presented in Table 2 and Table 3. Table 2 compiles the outcomes of studies using honey as a whole food, whereas Table 3 gathers data from studies employing honey-derived extracts. Furthermore, a schematic overview of the proposed mechanisms of action is provided in Figure 1, integrating current evidence on how honey may interact with key pathological processes in AD.

### 3.1. Oxidative Stress

Oxidative stress has been widely implicated in the pathogenesis of age-related diseases like AD, contributing to neuronal damage through the accumulation of reactive oxygen species (ROS) and impaired antioxidant defenses. The beneficial activity of different types of honey or their extracts against oxidative stress in the context of the nervous system has been evaluated using both in vitro and in vivo models. In terms of extracts, those obtained from multifloral and chestnut honeys have been evaluated in cell culture models. In N13 microglial cells, lipopolysaccharide (LPS)-induced ROS production was significantly attenuated by pretreatment with 0.5 and 1 μg/mL of a flavonoid-rich extract of multifloral honey [19]. Similarly, the ethyl acetate fraction of chestnut honey extract reduced intracellular ROS levels in HT22 neuronal cells exposed to glutamate in a dose-dependent manner [50]. With respect to whole honey, both multifloral and manuka varieties have been assessed in cellular models. An attenuation of oxidative stress was found in an astrocyte cell culture damaged with H_2_O_2_, where honey treatment provided dose-dependent protection, with the 1% (*v*/*v*) dose showing the most significant effect on cell survival [10]. manuka honey has also demonstrated ROS-reducing effects, as observed in LPS-injured RAW 264.7 macrophages. In addition, this treatment reduced oxidative damage to lipids, proteins, and DNA, as evidenced by decreased thiobarbituric acid reactive substances (TBARS) and protein carbonyl levels, as well as reduced expression of the DNA repair enzyme 8-oxoguanine DNA glycosylase (OGG)1 [30].

In *Caenorhabditis elegans*, the increase in the ROS content caused by 2,2′-azobis (2-amidinopropane) dihydrochloride (AAPH) was significantly decreased by 100 mg/mL of manuka honey [17] and the same concentration of avocado honey [18], with the effect of the avocado honey being greater. Results in the same sense were described for coffee honey (*Apis cerana*) treatment at 1% (*v*/*v*) applied to transgenic fly larvae (*Drosophila melanogaster*). The analysis of brain homogenates revealed a lower ROS content in treated flies comparing to the control group, which is indicative of an attenuation of oxidative stress in the central nervous system [32]. *C. elegans* [52] and *D. melanogaster* [53] are widely used in in vivo models for the screening and evaluation of potential health benefits of natural or synthetic compounds. Regarding the nematode model, *C. elegans* shares many conserved biochemical pathways with mammals. Genes associated with AD in humans have orthologs in *C. elegans*, including those involved in amyloid protein precursor (APP) processing and tau protein regulation. Moreover, since the complete neuronal connectivity of the worms has been fully mapped, it provides a valuable model for studying learning and memory impairments associated with AD [52]. Similarly, *D. melanogaster* has proven useful for modeling AD, as the expression of disease-related human gene products, such as tau and Aβ_42_, leads to observable and quantifiable phenotypes in flies. This makes *D. melanogaster* a practical model for investigating specific pathological features of AD. Additionally, more complex behavioral and cognitive assays can also be conducted in this model [53]. However, despite the numerous advantages of these non-vertebrate models, such as short lifespan, genetic manipulability, and cost-effectiveness, their findings cannot be directly extrapolated to humans. Therefore, results obtained in *C. elegans* and *D. melanogaster* should be further validated in higher-order animal models, such as rodents, and ultimately in clinical trials to assess translational relevance.

Experiments carried out in in vivo models of neurological damage are consistent with the results already mentioned in cells and invertebrates. Several toxic agents, such as Aβ_1-42_, LPS, kainic acid (KA), AlCl_3_, lead (Pb), or even a high-fat diet (HFD) treatment, are used in order to induce inflammation, oxidative stress, or cognitive impairment. The total antioxidant capacity (TAC) and TBARS levels, which initially were affected by AlCl_3_ [33] or KA [34,35] in animal models, were improved by the treatment with honey syrup [33] and tualang honey [34,35], respectively. Interestingly, in latter study, authors administered the treatment as honey silver nanoparticles [35]. In addition, tualang honey, a wild polyfloral honey produced by *Apis dorsata*, increased the number of viable cells in the cortex of rats injured with KA, a well-known neurotoxic agent [54]. The LPS-induced oxidative stress was ameliorated by honey treatment in rats, as manifested the lower malondialdehyde (MDA) levels in the brain [36,38]. However, the MDA levels were not affected by kelulut honey treatment in the hippocampus of Sprague Dawley rats injured with Aβ_1-42_ (AD model) [41], which could be related to the type of honey. On the other hand, treatment with chestnut honey for 10 weeks decreased the ROS and nitrite content in the brain of C57BL/6 mice injured with HFD. Authors evidenced that HFD was able to induce activation of amyloidogenic pathways, neuroinflammation and neurodegeneration; therefore, it is a suitable model for the evaluation of the natural products’ effects on AD [13]. Regarding lead exposure, it has been reported to cause reduced locomotor and exploratory activity, increased anxiety-like behavior, memory impairment, enhanced lipid peroxidation, and diminished antioxidant enzyme activity. In a study using male Wistar rats exposed to lead acetate, concomitant oral administration of honey for 28 days resulted in improved memory performance. However, the treatment did not significantly alter MDA levels in the brain [37]. On the other hand, aged male Sprague Dawley rats subjected to noise-induced stress were used to evaluate the effects of tualang honey. The animals received oral administration of tualang honey for 14 days prior to the stress procedure and continuously during the 14-day stress exposure period. This treatment improved both short- and long-term memory performance and significantly reduced MDA levels in the brain [39]. Along the same line, tualang honey administered to Sprague Dawley rats subjected to stresses increased the TAC and reduced-oxidized glutathione (GSH:GSSG) ratio in the brain, while reducing the TBARS and GSSG levels [40].

Regarding antioxidant defense, the activation of nuclear factor erythroid 2-related factor 2 (Nrf-2) in various neurodegenerative models promotes the expression of antioxidant enzymes, thereby reducing oxidative stress and exerting neuroprotective effects. In this context, one study evaluated the effect of a chestnut honey extract in HT22 cells exposed to glutamate-induced toxicity. The results showed that pretreatment with the extract significantly upregulated the expression of heme oxygenase-1 (HO-1) and the catalytic subunit of glutamate–cysteine ligase (GCLC). Additionally, a trend toward increased nuclear translocation of Nrf-2 was observed following treatment with the extract, although this change did not reach statistical significance [50]. Similarly, manuka honey demonstrated antioxidant effects in RAW 264.7 macrophages exposed to LPS-induced oxidative stress. Treatment with 8 mg/mL for 24 h, in the presence of 1 μg/mL LPS, significantly reduced nitrite accumulation and increased intracellular GSH levels. Furthermore, the treatment enhanced the activity of glutathione reductase (GR) and glutathione S-transferase (GST), and upregulated the protein expression of Nrf-2, Keap1, catalase (CAT), and superoxide dismutase (SOD) [30]. In line with these findings, Cianciosi et al. [31] evaluated the effects of Italian chestnut and eucalyptus honeys in RAW 264.7 macrophages injured with LPS. Pretreatment with honey followed by stimulation with LPS significantly increased intracellular GSH levels and glutathione peroxidase (GPx) activity, while GR and GST activities remained unchanged. Notably, ROS levels and Nrf-2 gene expression were not significantly affected [31]. The specific composition of the chestnut and eucalyptus honeys used, including their polyphenolic profile and antioxidant capacity, may selectively modulate certain enzymatic pathways while leaving others unaffected, which could explain the differences between manuka and those honeys in the same experimental models. Furthermore, the timing and duration, as well as the dose applied, may not have been set optimally to trigger a full Nrf-2 activation or reduce ROS accumulation.

In other experimental models, authors reported that manuka [17] and avocado [18] honey treatments led to a decrease in SOD-3 expression in a *C. elegans* transgenic strain, accompanied by a reduction in heat shock protein (HSP)-16.2 expression, while maintaining GST-4 enzyme expression [17,18]. Interestingly, manuka honey did not affect the expression of Skinhead (SKN)-1/Nrf-2 and dauer formation (DAF)-16/Forkhead box class O (FOXO) transcription factors [17]. In contrast, avocado honey treatment resulted in an upregulation of DAF-16/FOXO and a downregulation of SKN-1/Nrf-2 expression [18]. These divergent responses likely reflect differences in the phytochemical compositions of the two honeys, suggesting that each honey type activates distinct antioxidant signaling pathways to confer cellular protection. Both honeys contain substantial amounts of compounds such as o-vanillin and ellagic acid, which may underlie their shared effects on several antioxidant markers. However, unique bioactive constituents distinguish each honey: manuka honey is enriched in flavonoids and phenolics including epicatechin, naringenin, and quercetin [17], whereas avocado honey contains notable levels of ferulic acid and isoflavones like formononetin [18].

On the other hand, tualang honey and its phenolic extract increased the depleted levels of CAT and GPx in the LPS-injured rat brain [38]. In addition, the whole honey increased the levels of the antioxidant enzymes SOD and GR [38], and reduced glutathione (GSH) content [36]. Neuroprotective effects were further supported by an increased number of Nissl-positive cells in the hippocampus, indicating preserved neuronal integrity [38]. Similarly, in a lead-induced neurotoxicity model, honey administration restored SOD, GSH, and GST activity in rat brain [37]. In a noise-induced stress model, tualang honey improved SOD activity, which correlated with decreased MDA levels and better memory performance. However, no changes were observed in GPx, GR, or CAT activity under these conditions [39]. In addition, treatment with honey syrup for 45 days attenuated the oxidative effects of AlCl_3_, restoring SOD, CAT, and GPx activity [33]. In contrast, a 28-day treatment with kelulut honey failed to modify SOD1 levels in the hippocampus of Sprague Dawley rats subjected to Aβ_1–42_-induced injury (AD model), which is consistent with its lack of effect on oxidative damage markers in the same study [41]. Further evidence from a KA-induced excitotoxicity model supports the antioxidant potential of tualang honey. In this study, oral administration of silver nanoparticles synthesized with tualang honey significantly reduced brain nitrogen oxides (Nox) levels and increased CAT activity and GSH content [35].

As summarized in Table 1 and Table 2, the dosages and treatment protocols vary greatly across the experiments, making it difficult to directly compare the effects of different honeys and determine which variety is more effective in mitigating oxidative stress. The studies used doses ranging from 10 mg/kg/day over extended periods (e.g., 45 days) to 1 g/kg administered multiple times over short intervals (e.g., five times every 12 h). Furthermore, experimental models and the agents used to induce oxidative stress also differ. Nonetheless, the overall findings suggest that various types of honey may serve as potential therapeutic agents against oxidative stress and neurodegeneration. They appear to reduce ROS and markers of oxidative damage while enhancing antioxidant defense mechanisms. Thus, in this sense, honey may represent a promising dietary approach for the management of oxidative stress in the neurological context of AD.

Considering dose extrapolation from rodents to humans, the method based on body surface area is commonly used [55]. This approach calculates the human-equivalent dose (HED) by applying species-specific conversion factors. For example, a dose of 10 mg/kg administered to rats would correspond to approximately 1.6 mg/kg in humans. For a standard 60 kg adult, this results in a total daily dose of around 96 mg. Similarly, a high dose such as 1 g/kg in rats translates to 161 mg/kg in humans, which equals 9.6 g per day for a 60 kg adult, which is still within the range of typical daily honey intake. Therefore, the doses used in several animal studies appear to be physiologically and nutritionally relevant for humans.

### 3.2. Mitochondrial Dysfunction

The primary mitochondrial cascade hypothesis proposes that mitochondria drive the pathogenesis of AD. Mitochondrial dysfunction drives Aβ production and tau deposition, affecting the maintenance of optimal neuronal and synaptic function. Targeting the mitochondria and/or associated proteins may hold promise for new therapeutic strategies for AD, and for providing meaningful clinical benefit. One therapeutic approach is to restore mitochondrial function and reverse oxidative stress by using antioxidants [56]. Authors evaluated the effect of an extract of chestnut honey on glutamate-exposed neurotoxicity in HT22 cells. The glutamate exposure rapidly changed the mitochondrial membrane potential of neurons, leading to a significant loss. Alternatively, the treatment with the honey extract prevented glutamate-induced mitochondrial membrane potential loss at concentrations of 500 and 750 μg/mL, in a concentration-dependent manner [50].

### 3.3. Neuroinflammation

Neuroinflammation is increasingly recognized as a key driver in the progression of pathology, as sustained activation of glial cells and pro-inflammatory signaling contribute to neuronal dysfunction and degeneration. Nuclear factor kappa B (NF-κB) plays a central role in the inflammatory response by promoting the expression of pro-inflammatory cytokines and mediators such as tumor necrosis factor-alpha (TNF-α), interleukins (e.g., IL-1β, IL-6), cyclooxygenase-2 (COX-2), and inducible nitric oxide synthase (iNOS) [51]. The induction of iNOS expression during the inflammatory process catalyzes the conversion of L-arginine into nitric oxide (NO) [43]. Conversely, Nrf-2 exerts anti-inflammatory effects not only by enhancing antioxidant defenses but also by inhibiting the NF-κB signaling pathway, thereby suppressing the production of pro-inflammatory molecules [51]. In the context of neurodegeneration, chronic activation of NF-κB contributes to sustained neuroinflammation, neuronal damage, and disease progression. Several studies have shown that honey and its phenolic compounds can inhibit NF-κB activation by reducing its nuclear translocation or interfering with upstream signaling pathways, ultimately leading to decreased expression of inflammatory mediators.

Some studies that showed reduced ROS content also reported decreased inflammatory markers. For instance, the anti-inflammatory activity of various Algerian honeys was evaluated in vitro, and the samples exhibited a significant inhibitory effect on heat-induced bovine serum albumin (BSA) denaturation, a property related to their antioxidant capacity [42]. Kelulut honey has also demonstrated anti-inflammatory properties in RAW 264.7 cells stimulated with LPS. Specifically, concentrations of 0.5% and 1% (*v*/*v*) led to a reduction in NO levels and iNOS expression, although COX-2 expression was not altered [43]. A similar experimental model was used by Gasparrini et al. [30] to evaluate the anti-inflammatory activity of manuka honey. Their results showed that treatment with manuka honey led to a downregulation of NF-κB, iNOS, TNF-α, and IL-1β gene expression, along with a decrease in toll-like receptor (TLR)-4, NF-κB, p-Iκ-Bα, and iNOS protein levels. Additionally, the treatment reduced the protein expression of pro-inflammatory cytokines TNF-α, IL-1β, and IL-6, while increasing the expression of the anti-inflammatory cytokine IL-10 [30]. Treatment with chestnut or eucalyptus honey decreased NO levels in the macrophage cell line [31].

In the same cellular model, the anti-inflammatory effects of methanolic extracts of saffron and eryngium honeys were also investigated. RAW 264.7 macrophages were pretreated with saffron or eryngium honey extracts, and both treatments significantly reduced NO production and downregulated NF-κB and TNFSF9 gene expression. Additionally, saffron honey decreased IL-6 expression, while eryngium honey upregulated Nrf2 gene expression, suggesting activation of the antioxidant response pathway [51]. Similarly, the mRNA expression of pro-inflammatory markers was reduced in a dose-dependent manner by a flavonoid extract from multifloral honey (0.5 and 1 μg/mL) in an LPS-injured N13 microglial cell line. More specifically, TNF-α, IL-1β, and iNOS mRNA levels, and iNOS protein levels, were decreased by the treatment. Therefore, the extract is a potent inhibitor of microglial activation and a potential agent for neurodegenerative diseases involving inflammation [19].

In vivo experiments confirmed the anti-inflammatory activity of honeys in the neurological context. Tualang honey ameliorated the increase in TNF-α and IL-1β levels induced by kainic acid in different cerebral areas in rats. The glial fibrillary acidic protein (GFAP) and the allograft inflammatory factor 1 (AIF-1) levels were also significantly reduced by the treatment, as well as the levels of the enzymes COX-2 and 5-lipoxygenase (5-LOX). Thus, this honey reduced neuroinflammation in different brain regions [45]. The LPS-induced impairment of memory was improved by honey in Wistar rats [36] and by the honey and its extract in Sprague Dawley rats [57]. Additionally, locomotion activity was increased and the levels of the inflammatory markers TNF-α and IL-6 were reduced by treatment, as well as the nitrite levels [36]. NO is highly unstable and rapidly converts into nitrate and nitrite; therefore, nitrite levels are commonly assessed as an indirect yet stable indicator of NO production [51]. Similarly, chestnut decreased the HFD-induced gene expression of TNF-α as well as COX-2 and iNOS protein levels in mice [13], and stingless bee honey reduced the TNF-α levels in the brain of rats injured with HFD [44]. In contrast, kelulut honey did not alter the NF-κB levels in the hippocampus of rats injured with Aβ_1-42_ [41]. This absence of effect may be related to the specific properties of kelulut honey, which also failed to influence other parameters evaluated, in contrast to other honey types that showed positive outcomes. Alternatively, it may be due to the modulation of biological pathways not assessed in the study.

As previously mentioned, the assays conducted to evaluate inflammatory markers in the context of neurological disease vary considerably in terms of the type of honey used, the methodologies applied, and the experimental models employed. These differences hinder direct comparisons and limit the ability to extrapolate the findings. Notwithstanding this, in summary, honey and its phenolic compounds exhibit strong anti-inflammatory properties and may represent very interesting therapeutic agents for the treatment of neurodegenerative diseases.

### 3.4. Apoptosis

Dysregulated cell death pathways contribute to the gradual loss of cognitive function, so neuronal apoptosis plays a central role in AD progression. Honey and its polyphenols were found to be useful in improving memory deficits and could act at the molecular level by involving in apoptotic activities [58]. Proapoptotic proteins include Bax and Bim, whereas Bcl-2, Bcl-xL, and Bcl-w are considered anti-apoptotic. In addition, the FAS ligand (FAS-L) has been involved in neuronal death and P-27 has been reported to promote neuronal apoptosis induced by the Aβ_1-42_ peptide [13].

Although the evidence on AD is very scarce, different types of honey and their extracts have shown effects on apoptosis. Extracts from chestnut honey [50], as well as from saffron and eryngium honeys [51], have been investigated in vitro. A pretreatment with an ethyl acetate fraction of chestnut honey (500 or 750 μg/mL) decreased the AIF and increased the Bcl-2 protein expressions, as well as increased the survival of HT22 cells injured with glutamate [50]. In RAW 264.7 macrophages stimulated with LPS, methanolic extracts of saffron and eryngium honeys reduced Bax gene expression and increased Bcl-2 gene expression at concentrations selected based on MTT viability results (3.4 and 6.8 µg/mL for saffron; 5.5 and 11 µg/mL for eryngium), indicating antiapoptotic potential in an inflammatory context [51].

In vivo, chestnut [13] and kelulut honey [41] have been demonstrated to exert an influence on the apoptosis process. Treatments with chestnut and kelulut honey decreased the number of apoptotic cells in the cortex of rodents injured with HFD [13] and Aβ_1-42_ [41], respectively. The effect was mediated by the downregulation of pro-apoptotic genes such as FAS-L, P27, and BIM, and the upregulation of the anti-apoptotic Bcl-2 gene. Additionally, treatment increased the brain-derived neurotrophic factor (BDNF) gene expression that previously was decreased by the HFD [13]. However, not all types of honey exerted a measurable impact. In one study, treatment with stingless bee honey in HFD-fed Wistar rats did not significantly alter the number of apoptotic cells in the brain [44].

These findings suggest that certain honey types may modulate apoptosis-related pathways, although further studies are needed to confirm these effects and clarify the specific molecular mechanisms involved.

### 3.5. Aβ Plaque Damage and APP Processing

As already mentioned above, the accumulation of Aβ peptides into extracellular plaques is one of the hallmark features of AD, disrupting synaptic function and triggering a cascade of neurodegenerative events. Amyloid precursor protein (APP) undergoes two alternative processing routes: the non-amyloidogenic pathway, involving α- and γ-secretases, results in soluble fragments that are subsequently degraded and cleared, whereas in the amyloidogenic pathway, β- and γ-secretases generate insoluble Aβ peptides, which aggregate to form extracellular amyloid plaques [5,6]. The beta-site amyloid precursor protein cleaving enzyme (BACE1, also known as β-secretase) is implicated in the amyloidogenesis of AD and considered a key drug target as its inhibition is expected to decrease the formation of amyloid aggregates. Some analyses have revealed that compounds present in honey, such as rutin, 3,4-dicaffeoylquinic acid, nemorosone, and luteolin, have high binding affinity to the BACE1 receptor, positioning them as promising therapeutic agents [59]. *C. elegans* transgenic strains were used by some authors to investigate the effect of manuka honey [17] and avocado honey [18] in a paralysis phenotype caused by Aβ_1−42_ expression. Concentration of 100 mg/mL dramatically delayed the paralysis process with both treatments [17,18]. The RNAi test revealed that the effect of manuka honey was mediated by HSP-16.2 and the SKN-1/Nrf-2 pathway [17]. HSP16.2 is a chaperone involved in the correct folding of unfolded polypeptides [5] and SKN-1 is an important antioxidant pathway. Furthermore, the Thioflavin T stain showed a reduction in Aβ deposits in treated worms [17].

Although the investigation into rodent models is scarce, the results are in line with those already presented. Honey was evaluated in a mix with other functional foods such as pomegranate and dates, and the treatment with 4 mL/kg of that mixture manifested an improvement in spatial memory and learning in an AD model based on male albino Wistar rats injected with Aβ_42_ [60]. Oxidative stress and inflammation have been closely associated with increased synthesis and accumulation of Aβ peptides. In rat models, administration of LPS led to elevated levels of Aβ_1−42_ and reduced Aβ_1−40_ in the hippocampus compared to controls, suggesting an enhancement of amyloidogenic processes. Treatment with tualang honey mitigated these alterations, resulting in a reversal of this pattern, and higher Aβ_1−40_ and lower Aβ_1−42_ levels relative to the LPS group, indicating its potential to counteract oxidative damage and amyloid aggregation [38]. Similarly, kelulut honey was shown to reduce Aβ_1−42_ accumulation in the dentate gyrus of rats subjected to Aβ_1−42_-induced neurotoxicity [41]. On the other hand, chestnut honey decreased the expression of genes related to APP generation and processing in mice having injured HFD [13]. Reducing the levels of Aβ is a prime and a promising therapeutic strategy for treating AD, and honey could be beneficial in that sense.

### 3.6. Hyperphosphorylated Tau Protein Damage

Tau protein, located in the cytoskeleton microtubules, is phosphorylated by the kinases that are activated in turn by the Aβ. That phosphorylated tau leaves the microtubule and forms intracellular aggregates, which are strongly associated with neuronal dysfunction and cognitive decline [4,5]. To the best of our knowledge, there are only four studies in which the relation between honey and tau protein is addressed. The *C. elegans* transgenic strain BR5706, which expresses pro-aggregant human tau protein in neurons, was treated with manuka [17] and avocado [18] honey and the mobility was evaluated. Intriguingly, the honey-treated worms exhibited worse parameters of movement than the non-treated group in both types [17,18]. The authors evaluated those parameters in the wild strain too, and the locomotion deterioration was observed again. Moreover, the honey hydrophilic extract exerted a similar effect to that of the vehicle [17]. Thus, the sugar content of manuka or avocado honey could affect the general mobility of the nematodes and not specifically the aggregation of tau protein, but more studies are needed in order to clarify this hypothesis.

Nevertheless, more promising results have been observed in animal models. Male Sprague Dawley rats subjected to Aβ_1-42_ -induced injured and treated with kelulut honey showed reduced p-Tau levels in the hippocampus [41]. Similarly, Terzo et al. [13] used a HFD-induced model in mice to evaluate the effects of chestnut honey on tau protein, reporting a downregulation of genes associated with tau expression. These findings suggest that honey may exert beneficial effects on tau pathology; however, further studies are necessary to validate and confirm these results.

### 3.7. Imbalance of Neurotransmitters and Elimination-Related Enzymes

In terms of neurotransmission, AD is characterized by profound impairments in the cholinergic, glutamatergic, gamma-aminobutyric acid (GABA)ergic, and monoaminergic systems, which are closely linked to cognitive and behavioral symptoms. In particular, reduced acetylcholine levels and elevated AChE activity are strongly associated with memory loss, while glutamatergic dysregulation contributes to excitotoxicity and neuronal death [4]. The enzyme inhibitory potential of various types of honey has been extensively investigated in vitro. Buckwheat honey and multi-floral honey stood out for their AChE and butyrylcholinesterase (BChE) inhibitory activity, respectively [46]. In addition, other varieties such as acacia, raspberry, thyme, and rosemary also exhibited significant inhibition of both enzymes [47]. Moreover, the AChE inhibitory activity of manuka honey has also been demonstrated [61]. The phenolic extract of some Algerian honeys [42] and acacia honey [48] exhibited a significant inhibition of AChE. Correlations were observed between those properties and the antioxidant capacity of the honeys [42]. The in vitro results of acacia honey were confirmed by in vivo experiments in Wistar rats. The treatment reduced the AChE activity in the brain and serum [48]. The AChE activity, which was increased by LPS induction, was attenuated by honey treatment, again in Wistar rats [36]. Along the same line, tualang honey treatment for 28 days (orally) decreased the AChE activity of aged rats [39]. Monoaminoxidase (MAO) is another enzyme involved in neurodegenerative diseases such as AD. Chestnut honey and other bee products were effective in inhibiting MAO activity in enzymes isolated from rat liver microsomes. That effect was related to the antioxidant capacity of the samples [49]. As manifested, honey can be a rich source of inhibitors for ChE and other enzymes, and, therefore, may play an important role in AD treatment. Thus, those results further support the potential neuroprotective properties of different honey types, and the effect should be further explored for the management of AD.

## 4. Limitations and Final Conclusions

This review highlights the potential neuroprotective properties of honey and honey-derived extracts against key pathological mechanisms involved in AD, including oxidative stress, inflammation, apoptosis, and Aβ or tau-related pathways. Nevertheless, the current body of evidence remains predominantly preclinical, which significantly limits the translational applicability of the findings.

One of the main limitations is the lack of clinical trials investigating the effects of honey in AD. To our knowledge, no randomized controlled human studies have been completed or are currently registered that directly evaluate honey as a therapeutic or preventive agent for this disease. Therefore, any extrapolation of the available data to human health must be interpreted with caution. Furthermore, a high degree of heterogeneity is observed across the included studies regarding the type of honey tested, animal species or cell lines used, doses administered, treatment duration, and outcome measures employed. Even when the same variety of honey is used (e.g., manuka or chestnut), its phytochemical composition can vary substantially depending on its geographic and botanical origin, harvest season, and processing method. These factors hinder the ability to draw robust, generalizable conclusions about the comparative efficacy of different honeys and make it hard to propose standardized dosage or therapeutic protocols at this stage. Additionally, many studies rely on invertebrate models (e.g., *C. elegans*) or murine organisms, which, while valuable for mechanistic exploration, limit the direct translatability of the results to humans. The doses used in animal studies are often very high (e.g., up to 1 g/kg/day) and may not be realistically achievable or acceptable in human dietary or therapeutic contexts. No attempts were made in the cited literature to convert these doses to human-equivalent doses, nor to address pharmacokinetic aspects such as the low oral bioavailability of polyphenols, which further complicates the clinical relevance of the findings.

To address the methodological quality and potential risk of bias in the included studies, appropriate tools were applied depending on the experimental model. For in vitro studies, the QUIN tool was used as a reference framework. Most studies showed a high risk of bias in recurrent aspects, particularly due to insufficient reporting on operator involvement, lack of blinding of outcome assessors, and inadequate standardization of measurement procedures. For in vivo studies, SYRCLE’s Risk of Bias tool was considered. Although many studies provided adequate details regarding animal characteristics and experimental conditions, frequent sources of bias included lack of allocation concealment, absence of blinding, and non-randomized outcome assessment.

Despite the aforementioned limitations, honey has consistently demonstrated the ability to reduce oxidative stress, enhance antioxidant defenses, decrease inflammation, and modulate enzymes involved in neurotransmission such as AChE. However, further research is needed to explore its role in other neurodegenerative mechanisms, including apoptosis, mitochondrial dysfunction, and the accumulation of pathological hallmarks such as amyloid-beta plaques and hyperphosphorylated tau protein. These processes are closely associated with neuronal damage and loss, and are fundamental drivers of AD progression, highlighting the need for further in-depth investigation. Among the types, avocado, manuka, chestnut, kelulut, and tualang honeys have been the most extensively studied, providing valuable insights into their distinct bioactive profiles and biological impacts. To conclude, honey holds promise as a functional food or as a source of phytochemicals for the development of nutraceuticals with potential use in the neurological context, although additional studies are required to draw robust conclusions. 

## Figures and Tables

**Table 1:** Main phenolic compounds identified in different types of honey.

Compound/Honey Type	AH	AVH	CH	COH	EH	KH	LH	MH	MFH	RH	THH	TH
Flavonoids												
Apigenin	X		X	X	X		X	X	X		X	X
Catechin	X			X			X	X	X			X
Chrysin	X	X	X					X	X	X	X	X
Epicatechin							X	X				
Galangin	X				X		X	X			X	
Genistein	X								X			
Isorhamnetin	X		X					X	X		X	
Kaempferol	X		X	X	X	X		X	X	X	X	X
Luteolin	X		X	X	X	X	X	X	X	X	X	X
Myricetin	X			X	X						X	
Naringenin	X			X		X		X				
Pinobanksin	X	X	X		X		X	X	X	X	X	
Pinocembrin	X		X		X		X	X	X	X	X	
Quercetin	X		X	X	X	X	X	X	X	X	X	X
Rutin	X			X		X	X		X			
Phenolic acids												
2-Hydroxycinnamic acid					X		X					X
Caffeic acid	X	X	X	X		X	X	X	X		X	X
Chlorogenic acid	X			X			X	X	X		X	X
Cinnamic acid			X	X	X	X	X	X	X		X	X
Ellagic acid		X	X						X			
Ferulic acid	X	X	X	X	X	X		X	X		X	X
Gallic acid	X		X	X		X		X			X	X
*p*-Coumaric acid	X	X	X	X		X	X	X	X	X	X	X
*p*-Hydroxybenzoic acid	X		X				X	X	X			
Rosmarinic acid		X								X		
Syringic acid	X	X			X	X	X	X	X		X	X
Vanillic acid	X		X									

Abbreviations and references for each honey type: Acacia Honey (AH): [11,14,15,22,24,26,28]; AVH (Avocado Honey): [18,27]; CH (Chestnut Honey): [20,27,28]; COH (Coffee Honey): [21,22,23]; EH (Eucalyptus Honey): [20,27]; Kelulut Honey (KH): [24,25]; Lavender Honey (LH): [20,28]; Manuka Honey (MH): [11,16,17,22]; MFH (Multifloral Honey): [19,26,27,28]; Rosemary Honey (RH): [11,20,27]; Thyme Honey (THH): [11,20,27]; Tualang Honey (TH): [11,15,16,24,25]. ‘X’ indicates the presence of the compound in that honey variety.

**Table 2:** Honey’s effects on AD features.

Model	Treatment	Dosage and Duration	Effects	Reference
Oxidative stress				
In vitro. Astrocyte cell culture injured with H_2_O_2_	Honey	1% (*v*/*v*) for 24 h followed by 100 μM H_2_O_2_ for 3 h	↑ Cell viability	[10]
In vitro. RAW 264.7 macrophages injured with LPS	Manuka honey	8 mg/mL honey with 1 μg/mL for 24 h	↓ ROS content ↓ TBARS and carbonyl protein levels ↓ Protein expression of OGG1 (DNA damage) ↑ GSH levels ↑ GR and GST activity ↑ Protein expression of Nrf-2, Keap-1, CAT and SOD	[30]
In vitro. RAW 264.7 macrophages injured with LPS	Chestnut and Eucalyptus honey	1 mg/mL Honey for 24 h followed by 1 µg/mL LPS for 24 h	= ROS content ↓ NO levels ↑ GSH levels ↑ GPx activity = GR and GST activity = Nrf-2 gene expression	[31]
In vivo. *C. elegans* worms injured with AAPH	Manuka honey	100 mg/mL for 48 h followed by 2.5 mM AAPH for 15 min	↓ ROS content	[17]
In vivo. *C. elegans* worms injured with AAPH	Avocado honey	100 mg/mL for 48 h followed by 2.5 mM AAPH for 15 min	↓ ROS content	[18]
In vivo. *C. elegans* transgenic strains	Manuka honey	100 mg/mL for 48 h	↓ SOD3 and HSP-16.2 expression = SKN-1/NRF2, DAF-16/FOXO, GST-4, and HSF-1 expression	[17]
In vivo. *C. elegans* transgenic strains	Avocado honey	100 mg/mL for 48 h	↑ DAF-16/FOXO expression ↓ SKN-1/NRF2, HSF-1, SOD-3 and HSP-16.2 expression = GST-4 expression	[18]
In vivo. *Drosophila melanogaster* dUbqn knockdown	Coffee honey	1% (*v*/*v*)	↓ Brain ROS content	[32]
In vivo. Balb/c mice injured with AlCl_3_ (model of AD)	Honey syrup	40 mg/kg/d (i.p.) AlCl_3_ followed by 500 mg/kg honey syrup (i.p.) for 45 days	↑ TAC ↓ TBARS = SOD activity ↑ CAT and GSH-Px activity	[33]
In vivo. C57BL/6 mice injured with AlCl_3_ (model of AD)	Honey syrup	40 mg/kg/d (i.p.) AlCl_3_ followed by 500 mg/kg honey syrup (i.p.) for 45 days	↑ TAC ↓ TBARS ↑ SOD, CAT and GSH-Px activity	[33]
In vivo. Male C57BL/6 mice injured with HFD	Chestnut honey	45 mg/d (orally) for 10 weeks	↓ ROS levels in brain	[13]
In vivo. Male Sprague Dawley rats injured with KA	Tualang Honey	1.0 g/kg (i.g.) five times every 12 h and 15 mg/kg KA (s.c.) 30 min after last dose of treatment	↑ Viable cells in cortex ↑ TAC in cortex ↓ TBARS levels in cortex	[34]
In vivo. Male Sprague Dawley rats injured with KA	Tualang Honey	10 or 50 mg/kg honey (p.o.) and 15 mg/kg KA (s.c.) 30 min after last dose of treatment	↑ TAC ↓ MDA and NOx levels in brain = Protein carbonyl levels in brain ↑ CAT activity ↑ GSH levels	[35]
In vivo. Male Wistar rats injured with LPS	Honey	250 µg/kg LPS (i.p.) for 7 days followed by 0.31 and 0.36 g/kg honey for other 7 days	↓ MDA levels in brain ↑ GSH levels in brain	[36]
In vivo. Male Wistar rats injured with lead acetate	Honey	1.5 mL/kg of honey concomitantly with 0.2% lead for 28 days (orally)	↑ Memory function = MDA levels ↑ SOD levels ↑ GST activity = CAT levels ↑ GSH levels	[37]
In vivo. Male Sprague Dawley rats injured with LPS	Tualang honey	200 mg/kg tualang honey (i.p.) for 14 days, 5 mg/kg LPS (i.p.) applied on day 4	↑ CAT and GPx levels ↑ SOD and GR levels	[38]
In vivo. Aged male Sprague Dawley rats injured with noise stress	Tualang honey	200 mg/kg bw/day (i.g.) 14 days prior to the stress procedure and continued for an additional 14 days during the stress exposure	↑ Short- and long-term memory ↓ MDA levels in brain ↑ SOD activity in brain = GPx, GR and CAT activity in brain	[39]
In vivo. Male Sprague Dawley rats subjected to stresses	Tualang honey	1 g/kg bw/twice daily (i.g.) and exposed to stress for 28 consecutive days	↑ TAC in brain ↓ TBARS levels in brain ↑ GSH:GSSG ratio in brain ↓ GSSG levels in brain	[40]
In vivo. Male Sprague Dawley rats injured with Aβ_1-42_ (AD model)	Kelulut honey	2.5 µg/µL Aβ_1-42_ (icv) 1 week before 1 g/kg bw/d honey (i.g.) for 28 days	= SOD1 and MDA levels in hippocampus	[41]
Neuroinflammation				
In vitro	Algerian honeys	IC_50_ = 1.72–7.43 mg/mL	↓ Activity of BSA denaturation induced by heat	[42]
In vitro. RAW 264.7 macrophages injured with LPS	Manuka honey	8 mg/mL honey with 1 μg/mL LPS for 24 h	↓ Nitrite levels ↓ NF-κB, iNOS, TNF-α and IL-1β gene expression ↓ TLR4, NF-κB, p-Iκ-Bα, iNOS protein expression ↓ TNF-α, IL-1β, IL-6 protein expression ↑ IL-10 protein expression	[30]
In vitro. RAW 264.7 macrophages injured with LPS	Chestnut and Eucalyptus honey	1 mg/mL honey for 24 h followed by 1 µg/mL LPS for 24 h	↓ Nitrite levels	[31]
In vitro. RAW 264.7 cell line injured with LPS	Kelulut honey	0.5 and 1% (*v*/*v*) honey for 2 h followed by the addition of 1 µg/mL LPS for another 22 h	↓ iNOS expression ↓ NO levels = COX-2 expression	[43]
In vivo. Male C57BL/6 mice injured with HFD	Chestnut honey	45 mg/d (orally) for 10 weeks	↓ Nitrite levels in brain ↓ TNF-α gene expression in brain ↓ COX-2 and iNOS protein levels in brain	[13]
In vivo. Male Wistar rats injured with HFD	Stingless Bee Honey	HFD for 8 weeks, followed by HFD with 10 mg/kg bw/d honey (i.g.) for another 8 weeks	↓ TNF-α levels in brain	[44]
In vivo. Male Sprague Dawley rats injured with KA	Tualang Honey	1.0 g/kg (i.g.) five times every 12 h and 15 mg/kg KA (s.c.) 30 min after the last dose of treatment)	↓ TNF-α and IL-1β levels ↓ GFAP and AIF-1 levels ↓ COX-2 and 5-LOX levels	[45]
In vivo. Male Wistar rats injured with LPS	Honey	250 µg/kg LPS (i.p) for 7 days followed by 0.31 and 0.36 g/kg honey for other 7 days	↓ Memory and motor impairment ↓ Nitrite levels in brain ↓ TNF-α and IL-6 levels	[36]
In vivo. Male Sprague Dawley rats injured with Aβ_1-42_ (AD model)	Kelulut honey	2.5 µg/µL Aβ_1-42_ (icv) 1 week before 1 g/kg bw/d honey (i.g.) for 28 days	= p-NF-κB levels in hippocampus	[41]
Apoptosis				
In vivo. Male C57BL/6 mice injured with HFD	Chestnut honey	45 mg/d (orally) for 10 weeks	↓ Number of apoptotic cells in cortex ↓ FAS-L, P27, and BIM gene expression ↑ BCL2 gene expression	[13]
In vivo. Male Wistar rats injured with HFD	Stingless Bee Honey	HFD for 8 weeks, followed by HFD with 10 mg/kg bw/d honey (i.g.) for another 8 weeks	= Number of apoptotic cells	[44]
In vivo. Male Sprague Dawley rats injured with Aβ_1-42_ (AD model)	Kelulut honey	2.5 µg/µL Aβ_1-42_ (icv) 1 week before 1 g/kg bw/d honey (i.g.) for 28 days	↓ Number of apoptotic cells in dentate gyrus, CA1 and CA3 areas	[41]
Aβ plaque damage and APP processing				
In vivo. *C. elegans* transgenic strain (CL4176, Aβ model)	Manuka honey	100 mg/mL for 72 h	↓ Aβ-induced paralysis phenotype ↓ Aβ deposits	[17]
In vivo. *C. elegans* transgenic strain (CL4176, Aβ model)	Avocado honey	100 mg/mL for 72 h	↓ Aβ-induced paralysis phenotype	[18]
In vivo. Male C57BL/6 mice injured with HFD	Chestnut honey	45 mg/d (orally) for 10 weeks	↓ Expression of genes related to APP generation and processing	[13]
In vivo. Male Sprague Dawley rats injured with LPS	Tualang honey	200 mg/kg tualang honey (i.p.) for 14 days, 5 mg/kg LPS (i.p.) applied on day 4	↓ Aβ_1−40_ levels ↑ Aβ_1−42_ levels	[38]
In vivo. Male Sprague Dawley rats injured with Aβ_1-42_ (AD model)	Kelulut honey	2.5 µg/µL Aβ_1-42_ (icv) 1 week before 1 g/kg bw/d honey (i.g.) for 28 days	↓ Aβ_1−42_ depositions in dentate gyrus area = Aβ_1−42_ depositions in CA1 and CA3 areas	[41]
Hyperphosphorylated tau protein damage				
In vivo. *C. elegans* transgenic strain (BR5706, tauopathy model)	Manuka honey	100 mg/mL for 72 h	↓ Mobility parameters	[17]
In vivo. *C. elegans* transgenic strain (BR5706, tauopathy model)	Avocado honey	100 mg/mL for 72 h	↓ Mobility parameters	[18]
In vivo. Male C57BL/6 mice injured with HFD	Chestnut honey	45 mg/d (orally) for 10 weeks	↓ Expression of genes related to tau protein	[13]
In vivo. Male Sprague Dawley rats injured with Aβ_1-42_ (AD model)	Kelulut honey	2.5 µg/µL Aβ_1-42_ (icv) 1 week before 1 g/kg bw/d honey (i.g.) for 28 days	↓ p-Tau levels in hippocampus	[41]
Imbalance of neurotransmitters and elimination-related enzymes				
In vitro	Polish honeys	Buckwheat honey (AChE inhibition 39.51%) Multi-floral honey (BChE inhibition 39.76%)	↓ AChE and BChE activity	[46]
In vitro	Acacia, raspberry, lavender, bean, buckwheat, aloe, heather, linden, eucalyptus, sunflower, goldenrod, linden, thyme, rape, rosemary, hawthorn, orange blossom honeys		↓ AChE and BChE activity	[47]
In vitro	Acacia honey	IC_50_ = 0.26% (*v*/*v*)	↓ AChE activity	[48]
In vitro: Enzyme isolated from rat liver microsomes	Chestnut honey	IC_50_ = 41.60 µg/mL	↓ MAO activity	[49]
In vivo. Male Wistar rats	Acacia honey	20% *v*/*v* (orally) for 1 week	↓ AChE activity in brain and in serum	[48]
In vivo. Male Wistar rats injured with LPS	Honey	250 µg/kg LPS (i.p) for 7 days followed by 0.31 and 0.36 g/kg honey for other 7 days	↓ AChE activity	[36]
In vivo. Aged male Sprague Dawley rats	Tualang honey	200 mg/kg bw/day (i.g.) 28 days	↓ AChE activity	[39]

Abbreviations: 5-LOX = 5-Lipoxygenase; AAPH = 2,2′-Azobis(2-amidinopropane) dihydrochloride; Aβ = amyloid β; AChE = acetylcholinesterase; AD = Alzheimer’s disease; AIF-1 = allograft inflammatory factor 1; APP = amyloid precursor protein; BChE = butylcholinesterase; BSA = bovine serum albumin; *C. elegans* = *Caenorhabditis elegans*; CAT = catalase; COX-2 = cyclooxygenase 2; DAF-16 = dauer formation 16; FOXO = Forkhead box class O; GFAP = glial fibrillary acidic protein; GPx = glutathione peroxidase; GR = glutathione reductase; GSH = reduced glutathione; GST = glutathione S transferase; HFD = high-fat diet; HSF = heat shock factor; HSP = heat shock protein; IC_50_ = half-maximal inhibitory concentration; icv = intracerebroventricular; i.g. = intragastric; IL = interleukin; i.p. = intraperitoneal; iNOS = inducible nitric oxide synthase; KA = kainic acid; LPS = lipopolysaccharide; MAO = monoamine oxidase; MDA = malondialdehyde; NF-κB = nuclear factor kappa B; Nrf-2 = nuclear factor erythroid 2-related factor 2; NO = nitric oxide; OGG1 = 8-oxoguanine DNA glycosylase 1; ROS = reactive oxygen species; s.c. = subcutaneous; SKN-1 = skinhead-1; SOD = superoxide dismutase; TAC = total antioxidant capacity; TBARS = thiobarbituric acid reactive substances; TLR = toll-like receptor; TNF-α = tumor necrosis factor alpha. Symbols: ↑ increase; ↓ decrease; = no significant change.

**Table 3:** Effects of honey’s extracts on AD features.

Model	Treatment	Dosage and Duration	Effects	Reference
Oxidative stress				
In vitro. N13 microglial cells injured with LPS	Flavonoid extract of multifloral honey	0.5 and 1 μg/mL for 30 min before 2.5 ng/mL LPS for 1 h	↓ ROS content	[19]
In vitro. HT22 cells injured with glutamate	Ethyl acetate Fraction of chestnut honey	250, 500, or 750 μg/mL for 24 h followed by 5 mM glutamate for 6 h	↓ ROS content ↑ HO-1 and GCLC protein expression	[50]
In vivo. Male Sprague Dawley rats injured with LPS	Phenolic extract of tualang honey	150 mg/kg extract (i.p.) for 14 days, 5 mg/kg LPS (i.p.) applied on day 4	↑ CAT and GPx levels	[38]
Mitochondrial dysfunction				
In vitro. HT22 cells injured with glutamate	Ethyl acetate Fraction of chestnut honey	500, or 750 μg/mL for 24 h followed by 5 mM glutamate for 6 h	↑ Mitochondrial membrane potential	[50]
Neuroinflammation				
In vitro. N13 cell culture injured with LPS	Flavonoid extract of multifloral honey	0.5 and 1 μg/mL, 30 min/6 h	↓ TNF-α, IL-1β, iNOS mRNA levels ↓ iNOS protein levels	[19]
In vitro. RAW 264.7 macrophages injured with LPS	Methanolic extract of saffron honey	3.4 or 6.8 µg/mL for 2 h, followed by 1 µg/mL LPS for 22 h	↓ NO production ↓ NF-κB, IL-6 and TNFSF9 gene expression	[51]
In vitro. RAW 264.7 macrophages injured with LPS	Methanolic extract of eryngium honeys	5.5 or 11 µg/mL for 2 h, followed by 1 µg/mL LPS for 22 h	↓ NO production ↓ NF-κB and TNFSF9 gene expression ↑ Nrf-2 gene expression	[51]
		Apoptosis		
In vitro. HT22 cells injured with glutamate	Ethyl acetate Fraction of chestnut honey	500, or 750 μg/mL for 24 h followed by 5 mM glutamate for 24 h	↓ Cell death ↓ AIF protein expression ↑ Bcl-2 protein expression	[50]
In vitro. RAW 264.7 macrophages injured with LPS	Methanolic extract of saffron and eryngium honeys	3.4 or 6.8 µg/mL (saffron) or 5.5 or 11 µg/mL (eryngium) for 2 h, followed by 1 µg/mL LPS for 22 h	↓ Bax gene expression ↑ Bcl-2 gene expression	[51]
Aβ plaque damage and APP processing				
In vivo. Male Sprague Dawley rats injured with LPS	Phenolic extract of tualang honey	150 mg/kg extract (i.p.) for 14 days, 5 mg/kg LPS (i.p.) applied on day 4	↓ Aβ_1−40_ levels ↑ Aβ_1−42_ levels	[38]
Imbalance of neurotransmitters and elimination-related enzymes				
In vitro	Phenolic extract of Algerian honeys	IC_50_ = 0.367–0.629 mg/mL	↓ AChE activity	[42]

Abbreviations: Aβ = amyloid β; AChE = acetylcholinesterase; AD = Alzheimer’s disease; AIF = allograft inflammatory factor; APP = amyloid precursor protein; CAT = catalase; GCLC = glutamate–cysteine ligase, catalytic subunit; GPx = glutathione peroxidase; HO-1 = heme oxygenase-1; IC_50_ = half-maximal inhibitory concentration; IL = interleukin; i.p.= intraperitoneal; iNOS = inducible nitric oxide synthase; LPS = lipopolysaccharide; NF-κB = nuclear factor kappa B; Nrf-2 = nuclear factor erythroid 2-related factor 2; NO = nitric oxide; ROS = reactive oxygen species; TNF-α = tumor necrosis factor alpha. Symbols: ↑ increase; ↓ decrease.
